# Epidemiology and healthcare access in Brazilian multiple sclerosis patients: insights from the BRANDO database

**DOI:** 10.1055/s-0045-1813263

**Published:** 2025-12-22

**Authors:** Alfredo Damasceno, Cintia Ramari, Carlos Tauil, Henry Koiti Sato, Dagoberto Callegaro, Maria Fernanda Mendes, José Artur Costa D'Almeida, Denise Sisterolli Diniz, Osvaldo J. M. Nascimento, Laura Parolin, Thiago Fukuda, Paulo Gama, Herval Soares Neto, Marco Lana-Peixoto, Giordani Rodrigues dos Passos, Rayllene Caetano, Kleber Cavalcante Santos, Caio César Diniz Disserol, Gabriel de Deus Vieira, Guilherme Diogo Silva, Eliana Cunha, Natália Talim, Mario B. Wagner, Milena Sales Pitombeira, Jefferson Becker

**Affiliations:** 1 Universidade Estadual de Campinas, Faculdade de Ciências Médicas, Departamento de Neurologia, Campinas SP, Brazil.; 2 University of Hasselt, Rehabilitation Research Center (REVAL), Faculty of Rehabilitation Sciences, Hasselt, Belgium.; 3 Universidade de Brasília, Brasília DF, Brazil.; 4 Instituto Neurológico de Curitiba, Departamento de Neuroimunologia, Curitiba PR, Brazil.; 5 Universidade de São Paulo, Faculdade de Medicina, Departamento de Neurologia, São Paulo SP, Brazil.; 6 Faculdade de Ciências Médicas da Santa Casa de São Paulo, São Paulo SP, Brazil.; 7 Hospital Geral de Fortaleza, Departamento de Neurologia, Fortaleza CE, Brazil.; 8 Hospital Geral de Goiás, Departamento de Neurologia, Goiânia GO, Brazil.; 9 Hospital Universitário Antônio Pedro, Departamento de Neurologia, Fortaleza CE, Brazil.; 10 Neurovie Neurologia e Saúde, Departamento de Neurologia, Joinville SC, Brazil.; 11 Universidade Federal da Bahia, Faculdade de Medicina da Bahia, Departamento de Neurologia e Psiquiatria, Salvador BA, Brazil.; 12 Universidade do Estado da Bahia, Departamento de Ciências da Vida, Salvador BA, Brazil.; 13 Pontifícia Universidade Católica de São Paulo, Faculdade de Ciências Médicas e da Saúde, Departamento de Neurologia, Sorocaba SP, Brazil.; 14 Hospital do Servidor Estadual de São Paulo, Departamento de Neuroimunologia, São Paulo SP, Brazil.; 15 Universidade Federal de Minas Gerais, Centro de Investigação de Esclerose Múltipla, Belo Horizonte MG, Brazil.; 16 Pontifícia Universidade Católica do Rio Grande do Sul, Hospital São Lucas, Porto Alegre RS, Brazil.; 17 Hospital das Forças Armadas, Brasília DF, Brazil.; 18 Universidade Federal do Paraná, Hospital das Clínicas, Departamento de Neurologia, Curitiba PR, Brazil.; 19 Universidade Federal do Rio Grande do Sul, Faculdade de Medicina, Departamento de Medicina Social, Porto Alegre RS, Brazil.; 20 Pontifícia Universidade Católica do Rio Grande do Sul, Faculdade de Medicina, Departamento de Medicina Social, Porto Alegre RS, Brazil.

**Keywords:** Multiple Sclerosis, Latin America, Epidemiology, Health Inequities, Nervous System Diseases, Demography

## Abstract

**Background:**

Multiple sclerosis (MS) is an inflammatory and neurodegenerative disorder whose prevalence varies across Brazil (from 15–27 cases per 100 thousand inhabitants), and the absence of an extensive national study limits the understanding of MS epidemiology in a nation as diverse as Brazil.

**Objective:**

To compare epidemiological data, including healthcare access, among people with MS across four Brazilian regions.

**Methods:**

Data from 2,974 Brazilian MS patients in the Collaborative Latin American Database for Multiple Sclerosis (BRANDO) were analyzed. We assessed demographic and clinical outcomes, as well as healthcare access, to elucidate regional differences.

**Results:**

The cohort was predominantly composed of female patients (72.5%) with MS onset at a mean age of 30.6 years. Regarding the regional differences, there was a lower predominance of female patients (68.7%; *p* = 0.003) in the Southeast, a higher rate of subjects of mixed ethnicity ( *p* < 0.001) in the Midwest (40.3%) and Northeast (63.7%), higher scores on the Expanded Disability Status Scale (EDSS) in the Northeast (4.0; *p* < 0.001), a higher prevalence of relapsing-remitting MS (RRMS) in the Southeast and Midwest (87%; *p* < 0.001), while the Northeast presented ( *p* < 0.001) the highest rates of primary progressive MS (PPMS) and secondary progressive MS (SPMS) (PPMS = 15.8%; SPMS = 18%). The Northeast presented the longest time (5.9 years; *p* < 0.01) from disease onset until MS diagnosis (range for the other regions = 1.9–3.7 years). And the Midwest showed the shortest time (2.1 years; *p* < 0.01) from disease onset until first access to disease-modifying therapies (DMTs; range for the other regions = 3.5–5.1 years).

**Conclusion:**

The present is the first nationwide epidemiological study on people with MS in Brazil. It underscores regional epidemiological variations and differences in healthcare access, advocating for tailored approaches in MS management and research.

## INTRODUCTION

Multiple sclerosis (MS) is the most common inflammatory disease of the central nervous system (CNS), 1 2 affecting over 2.8 million people worldwide. 2 It is a chronic and unpredictable disease that can impact mobility, cognition, and mood, and it is associated with lower quality of life. 3 This disease represents a significant personal and socioeconomic burden, given that its onset is typically around the age of 30, a period when individuals are actively contributing to society. 4

Given the significant impact MS has on patients and the abundance of affected individuals, numerous specialized medical centers for the treatment of neuroimmunological diseases, distributed across various countries, have conducted cohort studies to obtain a more detailed analysis of this complex disease and potentially optimize patient treatment. The United States of America (USA), France, Switzerland, and Sweden are some of the countries that conduct cohort studies following patients with MS. 5 6 For instance, in 2018, approximately 12 cohort studies with MS patients were being conducted in the USA, most of which are still ongoing. 5 Although Brazil has a lower MS prevalence compared to the aforementioned countries, it has a significant number of individuals with MS, with the disease occurring on average in 14 to 18 Brazilians per 100 thousand inhabitants, and in the Southern region, this prevalence can reach 27 per 100 thousand inhabitants. 7 8 9 Despite this, there is no MS cohort in Brazil comparing four different regions and including demographic and clinical data, which makes it difficult to understand the complexity of the disease in our country. 10

Another important factor influencing large cohort studies is the existence of established disease registries. These registries either gather new real-world data directly from patients, consolidate relevant data extracted from healthcare providers' electronic health record systems, or combine both approaches. Disease registries with high-quality data are required for many different purposes beyond direct patient care provision, such as quality improvement, safety monitoring, and research. 6 Examples of MS registries around the world are the Big Multiple Sclerosis Data (BMSD) network, 11 the North American Research Committee on Multiple Sclerosis (NARCOMS) registry, 12 and the MSBase registry, among others. Another important and independent multistakeholder initiative is the Multiple Sclerosis Data Alliance (MSDA), 6 acting under the umbrella of the European Charcot Foundation (ECF), to accelerate research insights for innovative care and treatment through better use of real-world data in MS. In Brazil, the Collaborative Latin American Database for Multiple Sclerosis (BRANDO) 13 acting under the umbrella of the Brazilian Committee for Treatment and Research in Multiple Sclerosis (BCTRIMS) and the Latin American Committee for Treatment and Research in Multiple Sclerosis (LACTRIMS) was launched in 2021. Currently, BRANDO collaborates with 6 countries, with 10 other countries in prospect, encompassing more than 40 centers and 190 collaborators.

In a country with a large and diverse population such as Brazil, understanding the prevalence and incidence of MS by analyzing retrospective data from a national MS disease registry, recognizing regional variations in clinical characteristics and risk factors related to the environment and lifestyle, can enhance strategies to manage MS. Regarding patient-centered care, precise epidemiological data can empower regional patient advocacy groups to campaign for better services, raise awareness about MS, and support affected individuals and their families. 14 Additionally, longitudinal epidemiological data can help understand the progression of MS, including the response to treatments. 15 For health equity, this can also highlight disparities in the occurrence and treatment of MS among different population groups, such as groups stratified by gender, race, socioeconomic status, and geographic location. 14 Thus, understanding the prevalence and incidence of MS in Brazil helps in the effective allocation of resources, including healthcare facilities, medical professionals, and funding for MS care and support services. 6 14

The aims of the current study are to describe and compare epidemiological data, including demographic and clinical outcomes, and healthcare access, among people with MS across four Brazilian regions.

## METHODS

The current is a descriptive and cross-sectional study with a retrospective analysis of epidemiological data from people diagnosed with MS from four regions of Brazil: Southeast, Northeast, South, and Midwest). The BRANDO project was approved by the Ethics Committee of Pontifícia Universidade Católica do Rio Grande do Sul (under CAAE:63172022.0.1001.5336), and the subjects provided informed consent.

### Participants

Data from 2,974 people with MS included in the BRANDO database were analyzed until April 2024. For the participant to be included in the study, the following inclusion criteria were required: diagnosis of MS according to the McDonald criteria 16 and follow up at one of the neuroimmunology reference centers participating in the BRANDO database; and registry in the BRANDO database with specific outcome measures relevant to the objectives of the current study.

### Data extraction

The current study only included data from the MS treatment centers that obtained approval from their local ethical committee and signed the agreement on data sharing and publication. A total of 14 MS treatment centers provided data from four Brazilian regions: South (3 MS centers), Southeast (7 MS centers), Midwest (2 MS centers), and Northeast (2 MS centers).

### Outcome measures

Demographic data such as sex, ethnicity (race, self-reported), age, age at disease onset, and treatment center were screened and extracted from the BRANDO database.

The MS-related clinical outcomes screened and exported from the database were disability status (according to the score on the Expanded Disability Status Scale, EDSS), relapse topography (that is, of the optic nerve, brain, posterior fossa, and spinal cord), MS phenotype (that is, relapse-remitting MS [RRMS], primary progressive MS [PPMS], and secondary progressive MS [SPMS]), and initial disease-modifying therapies (DMTs).

Regarding the outcomes related to healthcare access, the time frames in years were calculated regarding the following: disease onset until MS diagnosis, disease onset until first access to DMTs, and MS diagnosis until first access to DMTs.

### Statistical analysis

To analyze demographic and clinical data across different Brazilian regions, an initial descriptive analysis was performed. This included frequency counts for categorical variables such as sex and type of initial treatment, and measures of central tendency and dispersion for continuous variables such as age at disease onset and disability level. To compare these variables between regions, the Chi-squared test was used for the categorical data, and analysis of variance (ANOVA) or the Kruskal-Wallis test, for the continuous data, depending on the normality and homogeneity of the variables. Statistical significance was set at *p* < 0.05. The IBM SPSS Statistics for Windows (IBM Corp.) software, version 29.0.1.1, was used for the analysis.

## RESULTS

Table 1 presents the demographic and clinical characteristics of the cohorts across different Brazilian regions. As the sample size varied regarding the outcomes measured, the results presented in Tables 1 2 3 show the number (N) of data available for each outcome.

**Table 1:** Descriptive and clinical characteristics of the entire Brazilian cohort and regional subcohorts

	Brazil (N = 2,974)	South (N = 1,099)	Southeast (N = 1,128)	Midwest (N = 531)	Northeast (N = 216)
Sex* (N)	2,505	1,058	772	512	163
Female sex (%)	72.5	**68.7**	74.2	76.4	76.1
Ethnicity* (N)	1657	664	537	365	91
Caucasian (%)	75.8	**94.1**	**76.0**	**54.8**	**25.3**
Mixed-race (%)	18.5	**2.3**	**16.2**	**40.3**	**63.7**
African descendant (%)	5.3	3.3	**7.3**	4.7	**9.9**
Asian (%)	0.4	0.3	0.6	0.3	1.1
Age in years at onset** (N)	1,06530.8 (11.1)	33131.2 (29.9–32.5)	50230.4 (29.4–31.4)	14532.6 (31.0–34.1)	87 29.1 (26.9–31.2)
First application of the EDSS** (N): Score (0–10)	1,4722.8 (2.1)	4002.0 (0–9.0)	5712.0 (0–9.5)	367 **2.5 (0–9.0)**	134 **2.5(0–9.5)**
Last application of the EDSS** (N): Score (0–10)	8353.0 (2.1)	2302.0 (0–9.0)	2372.5 (0–8.5)	2712.5 (1.0–9.5)	97 **4.0 (0–9.5)**
MS henotype* (N)	2,664	1,099	818	531	216
Relapsing-remitting (%)	83.0	73.9	89.1	87.9	**63.9**
Secondary progressive (%)	9.7	8.6	4.6	9.0	**18.0**
Primary progressive (%)	5.4	5.7	5.6	2.3	**15.8**
**Initial treatment (N)**	2,664	1,099	818	531	216
Most used (%)	NTZ (17.9)	NTZ (16.2)	NTZ (21.5)	NTZ (15.5)	**GA (19.7)**

Abbreviations: EDSS, Expanded Disability Status Scale; GA, glatiramer acetate; MS, multiple sclerosis; NTZ, natalizumab.

Notes: The results for the Brazilian cohort are presented as mean and standard deviation values; the results for the regional cohort are presented as mean and 95%CI values, and median (minimum-maximum) for the EDSS score (which ranges from 0–10). Values in bold indicate significant differences among groups. Statistical tests used: *Chi-squared test; **Kruskal Wallis test; ***one-way analysis of varaiance; and the Tukey's honestly-significant Difference (HSD) post-hoc test.

**Table 2:** Description and prevalence of disease onset topography inserted in the BRANDO database for the entire Brazilian cohort and regional subcohorts

Topography		Brazil	South	Southeast	Midwest	Northeast	Comparison among groups
**Optic nerve**	N	821	241	240	39	45	*p* < 0.001
	%	48.8	37.8	57.5	71.8	42.2	
**Brain**	N	844	244	233	65	46	*p* < 0.001
	%	51.4	38.5	56.7	89.2	39.1	
**Posterior fossa**	N	729	225	175	27	46	*p* = 0.012
	%	37.6	31.1	40.0	51.9	52.2	
**Spinal cord**	N	812	241	223	43	49	*p* < 0.001
	%	53.6	43.6	61.9	76.7	44.9	

**Table 3:** Healthcare access timing by region in Brazil

Healthcare access	Brazil (N = 2,974)	South (N = 1,099)	Southeast (N = 1,128)	Midwest (N = 531)	Northeast (N = 216)	ANOVA among groups
Tome from disease onset until MS diagnosis**						Significance
N	772	219	378	77	98	F = 6.5
Years	3.65 (6.12)	3.7 (5.7)	3.4 (5.9)	**1.9 (3.3)**	**5.9 (8.5)**	*p* < 0.001
Time from disease onset until first DMTs**						
N	761	214	400	71	76	F = 7.39
Years	4.20 (5.90)	3.5 (5.0)	5.1 (6.4)	**2.1 (3.6)**	3.6 (6.2)	*p* < 0.001
Time from diagnosis until first DMTs**						
N	275	82	105	54	34	F = 2.6
Years	4.25 (11.46)	4.7 (18.9)	2.3 (4.6)	4.8 (6.3)	**8.3 (7.6)**	*p* = 0.05

Abbreviations: ANOVA, analysis of variance; DMTs, disease-modifying therapies; MS, multiple sclerosis.

Notes: Values refer to mean and standard deviation and values in bold indicate significant differences among groups. Statistical tests used: ***one-way ANOVA and the Tukey's honestly-significant Difference (HSD) post-hoc test.

### Demographics

As presented in Table 1 , in the South region, there was a lower predominance of female patients ( *p* = 0.003). Ethnicity was significantly different among regions ( *p* < 0.001): the Midwest and Northeast regions presented higher proportions of individuals of mixed ethnicity, while the Northeast and Southeast showed a higher presence of African descendants.

### Clinical outcomes

Regarding the clinical measures, MS-related outcomes and age at disease onset did not differ among the groups. The last EDSS score assessed indicated higher disability in the Northeast (median score of 4.0; *p* < 0.001) compared to other regions, where the median scores ranged from 2.0 to 3.0. The proportion of RRMS was higher in the Southeast and Midwest (87%; *p* < 0.001), whereas the Northeast exhibited the highest rates of PPMS (15.8%) and SPMS (18%; *p* < 0.001). Regarding the initial DMTs, glatiramer acetate was more commonly used in the Northeast compared to natalizumab, which was more prevalent in other regions.

Table 2 presents the topography regarding disease onset. For the entire Brazilian cohort, the most affected regions were the brain and spinal cord. The Southern and Southeastern cohorts showed more impairments in the spinal cord. However, the Southern cohort presented the lowest prevalence of impairment compared to all other regions. In contrast, the Midwestern sample showed a higher frequency of impairments, regardless of topography. The posterior fossa and spinal cord were the most affected regions in the Northeastern sample. The Pearson's Chi-squared test revealed significant differences among regions ( *p* < 0.001).

### Healthcare access

Concerning healthcare access, Table 3 shows that the Northeast presented the longest time from the disease onset to MS diagnosis compared to all other regions. The Midwest significantly presented the shortest time between disease onset and diagnosis, as well as between disease onset and first access to DMTs. Although without significant differences among the groups, the Southeast revealed the shortest time from MS diagnosis to the first access to DMTs, while the Northeast presented the longest time among the four regions, which was statistically significant.

**Figure 1:**
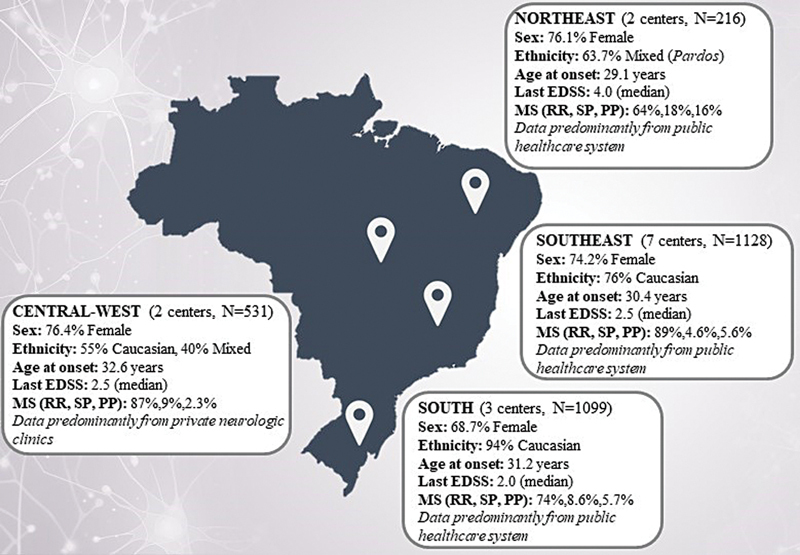
Note: The multiple sclerosis (MS) diagnoses followed the 2017 McDonald criteria; 16 MRI data entry was limited. Geographic distribution of study data in Brazil. The regional boxes summarize the demographics, Expanded Disability Status Scale (EDSS) scores, MS phenotype, and healthcare system characteristics.

Figure 1 presents the geographic distribution of study data across Brazil, with each regional panel summarizing demographic characteristics, EDSS scores, MS phenotypes, and relevant healthcare system features.

## DISCUSSION

The present study reveals significant regional disparities in the epidemiological and clinical profiles of MS patients across Brazil. Our findings highlight distinct patterns in sex and ethnicity distribution, as well as disability and healthcare access, underscoring the complexity of managing MS in a geographically-diverse country.

Variations in ethnicity are a relevant factor in Brazil, and studies 17 18 have suggested that the incidence and prevalence of MS may vary among people of different ethnicities, and that ethnicity may impact the severity of the disease and the rate of disability progression. The higher proportions of individuals of mixed ethnicity in the Midwest and Northeast, as well as the greater presence of African descendants in the Northeast and Southeast, reflect the expected ethnic composition of these regions based on the latest Brazilian census, 19 highlighting the country's diverse genetic background and warranting further investigation into the role of ethnicity in MS. Specifically, in the Northeast region, where a higher proportion of mixed-raced ( *pardos* , in Portuguese) individuals and African descendants were found, the disease disability status was significantly higher compared to that of the other regions, followed by a higher prevalence of progressive MS. In a review, Mallawaarachchi et al. 20 suggested that there is no consistent evidence to suggest that the relapse rate varies with ethnicity, but a number of studies 20 21 22 have found that people with MS of Black ethnicity accumulate disability at a faster rate than those of Caucasian ethnicity. However, studies investigating the association between ethnicity and MS course are scarce and warranted.

In the analysis of relapse topography within the Brazilian cohort, the brain and spinal cord emerged as the most frequently affected regions, though notable geographic variations were observed. Spinal cord involvement was particularly prevalent among patients from the South and Southeast regions, whereas individuals from the Midwest exhibited the highest overall prevalence of lesions across both brain and spinal areas. Conversely, the Northeastern cohort showed a predominance of lesions in the posterior fossa and spinal cord, potentially accounting for the higher levels of disability documented in this region. This observation is supported by findings made by Hartmann et al., 23 who examined the relationship between lesion location and clinical disability, underscoring the substantial impact of posterior fossa and spinal cord lesions on functional impairment. Lesions to the posterior fossa—which encompasses the brainstem and cerebellum—and the spinal cord are consistently associated with more severe disability in individuals with MS, in contrast to lesions located in other brain regions. 23 24

A notably high proportion of cases of relapse in our sample involved the brain (51.4%), which contrasts with reports from other countries, in which this figure is typically below 25%. This discrepancy warrants further investigation into the possible underlying causes. However, it is important to acknowledge that the retrospective design of the current study, which relied on existing database records, may have influenced the findings. Specifically, cases with MRI-confirmed topography may have been preferred for entry into the database over those based solely on clinical evaluation, potentially introducing a selection bias for this outcome. Additionally, in a systematic review, Nathoo et al. 25 examined differences in the MRI scans of people with MS of diverse racial and ethnic groups. They 25 found that research in diverse populations remains limited, but it suggests a trend in which African Americans with MS tend to show more severe MRI findings (such as greater lesion burden and significant atrophy) compared to Caucasian patients.

Disparities in the time from onset to diagnosis were shown by the fact that Northeast region presented the longest time from disease onset (that is, first relapse or symptom) to MS diagnosis compared to other regions. In contrast, the Midwest region presented the shortest time between disease onset and diagnosis, as well as between disease onset and access to DMTs. It is important to acknowledge that the present study employed a retrospective design, and the MS diagnoses may not have been uniformly based on the 2017 revision of the McDonald criteria. 16 In some cases, alternative diagnostic approaches, such as confirmation through a second clinical event or adherence to the 2010 McDonald criteria, may have been applied. Additionally, regional disparities in healthcare access represent a significant contextual factor. In the Northeast, MS treatment centers were predominantly part of the public healthcare system, whereas the data from the Midwest region were largely derived from a private neurological clinic contributing to the BRANDO database. Such differences in healthcare infrastructure and patient populations may have influenced diagnostic practices and timelines, potentially contributing to regional variation in the time until diagnosis.

According to Solomon et al., 26 a variety of contextual factors related to the affected individual, the health system, and policies contribute to delays in the diagnosis of MS globally. Their study 26 identified significant barriers, particularly in middle- and low-income countries, which include a lack of healthcare professionals—especially neurologists and MS specialists—and insufficient access to essential medical equipment and diagnostic tests, such as MRI scanners. Additionally, the cost of the recommended testing and the need for travel to MS centers often prevent individuals suspected of having MS from completing the necessary diagnostic procedures. These findings underscore the critical role of healthcare disparities in contributing to potential delays in MS diagnoses. In fact, the longest delay for MS diagnosis was observed in the Northeast, which has the lowest number of neurologists per 100 thousand inhabitants 27 among the 4 regions represented in our sample. Furthermore, the shortest and longest times from disease onset until MS diagnosis were observed in the Midwest and Northeast regions respectively, coinciding with the regions that present the highest and lowest numbers of MRI scanners in use per 100 thousand 28 respectively.

### Limitations and future perspectives

The current study is the first nationwide investigation of MS epidemiological data in Brazil. However, it has certain limitations. First, the sample size varied substantially regarding different outcome measures, with the number of available data inversely proportional to the complexity of the measure (such as disease onset topography). Another limitation is the variation in sample size across different Brazilian regions, underscoring the need for a task force to include more data from patients in the Northeast. We also highlight that the North region was not included due to a lack of data in the BRANDO database. Future studies could provide substantial information (including data on healthcare access) by including this region, given the significant contribution of Brazil's indigenous populations to the ethnicity of the North region.

Given that the BRANDO database was launched in 2021, the absence of consistent, comprehensive, and complex epidemiological MS data from Brazil still limits an understanding of the impact of ethnic differences and healthcare access on MS in the country. Additionally, globally, diverse data from Brazil are often not included in studies addressing ethnic disparities and healthcare access in relation to MS incidence, prevalence, disease severity and progression, and the efficacy of DMTs. 20

The present study reinforces the importance of systematically collecting and analyzing MS data in Brazil, rather than focusing solely on regional studies without broader comparisons. By highlighting disparities in disease presentation, healthcare access, and diagnosis timelines, our findings emphasize the need for neurologists to contribute patient data to MS databases, ensuring more comprehensive and reliable epidemiological studies. High-quality, descriptive data are essential to understand the true burden of MS in Brazil, identifying regional challenges and guiding healthcare policies. Expanding the BRANDO database with more representative data, particularly from underrepresented regions, will improve the accuracy of epidemiological studies and support better-informed decision-making to address disparities in MS care across the country.

In conclusion, the current study underscores the importance of recognizing and addressing regional disparities in MS epidemiology, clinical presentation, and healthcare access in Brazil. However, further research is needed to explore the underlying causes of these disparities, including mechanistic studies and correction for ethnicity and socioeconomic factors, to develop strategies to mitigate their impact.
